# Different immunotherapeutic combinations enhance specific T cell immune responses against leukemic cells, as well as leukemic progenitor cells, in acute myeloid leukemia

**DOI:** 10.1038/s41375-025-02764-7

**Published:** 2025-10-27

**Authors:** Jochen Greiner, Patrick J. Schuler, Hubert Schrezenmeier, Johanna Weiss, Christiane Bulach, Marlies Goetz, Barbara-ann Guinn

**Affiliations:** 1 Department of Internal Medicine, Diakonie Hospital Stuttgart, Stuttgart, Germany; 2 Department of Internal Medicine III, University Hospital Ulm, Ulm, Germany; 3 Department of Otorhinolaryngology (ENT), University Hospital Heidelberg, Heidelberg, Germany; 4 Institute of Transfusion Medicine, University of Ulm and German Red Cross, Ulm, Germany; 5 Centre for Biomedicine, Hull York Medical School, University of Hull, Hull, UK

**Keywords:** Immunotherapy, Acute myeloid leukaemia, Cell death and immune response, Haematopoietic stem cells, Preclinical research

## Abstract

Immunotherapeutic approaches have become increasingly important in cancer therapy, including for patients with acute myeloid leukemia (AML). Despite being shown to be effective in the context of stem cell transplants for almost 50 years, further improvements are required to prevent relapse and its associated morbidity. The therapeutic use of immune checkpoint inhibition in AML is still under debate. We have shown some positive effects of it on cancer control ex vivo. We found that anti-programmed death-1 (PD-1) antibodies in combination with azacitidine (AZA) had the most pronounced effect on T-cell activation and control of leukemic progenitor/stem cell growth. We identified which leukemia-associated antigen (LAA) stimulated the largest IFNγ immune response by T cells from AML patients with and without the nucleophosmin 1 (NPM1) mutation and which immunotherapeutic strategy, either alone or in combination with the immune checkpoint anti-PD-1, could enhance immune responses against leukemic cells. Anti-PD-1 with AZA had a particularly strong effect with a mean colony reduction of 56%. Taken together, combinations of immunotherapeutic approaches increase antigen-specific immune responses against leukemic cells but also leukemic progenitor/stem cells. The combination of LAA-peptides with anti-PD-1 antibody and one further immunotherapeutic could be an interesting option for further clinical studies.

## Introduction

Acute myeloid leukemia (AML) is a form of cancer that affects the bone marrow and is characterized by an abnormal growth and interruption in the differentiation of myeloid progenitor cells. AML primarily affects older individuals, a population that often lacks the physical strength for intensive treatment. While chemotherapy is the standard treatment for AML, alone it is insufficient at preventing the high rate of relapse among adults [1]. The 5-year survival rate for patients with AML is still poor, underscoring the urgent demand for innovative and synergistic treatments [2]. In recent years, there has been a growing emphasis on finding effective immunotherapy methods for AML, specifically those that target leukemia cells and their precursors. Given the complexity of AML, it may be beneficial to combine various immunotherapy approaches in a strategic manner to prevent escape mechanisms and limit potential toxicity [3–5].

Immunotherapeutic options, which are now commonly used in AML treatment, such as allogeneic hematopoietic stem cell transplantation and donor lymphocyte infusion, have shown significant success but also carry risks [6, 7]. On the other hand, other immunotherapeutic approaches such as immunotherapies with vaccines, bi-/tri-specific antibodies, hypomethylating agents, and CAR-T-cell treatment, to mention only a few, are still in the earlier stages of development and require further improvement [8–10].

Azacitidine (AZA) is a hypomethylating agent. It binds to DNA methyltransferases, enzymes responsible for the attachment of methyl groups to DNA, thereby inhibiting them. This enables AZA to cause demethylation of DNA, which leads to the reactivation of silenced genes. AZA can be used to enhance the T cell recognition of tumor cells, modulate the expression of immune checkpoints and stimulate anti-leukemia T cell activity [11]. In AML, novel drugs used in combination with chemotherapy, or as alternatives for patients who are not eligible for intensive treatment, are becoming increasingly employed. Combinations such as AZA with venetoclax, or ivosidenib with AZA, are already established as standard treatment options. Additionally, emerging therapies like FLT3 inhibitors, menin inhibitors, and immunomodulatory agents are showing promising results when used in double or triple combination regimens. Immunotherapies also have the potential to be integrated into these combination treatment strategies [12, 13].

Targeted immunotherapy in cancer treatment has become ever more important in recent years. The efficacy of immunotherapeutic approaches such as immune checkpoint inhibitors [14, 15], chimeric antigen receptor (CAR) T cells [16] or bi-specific T cell activating antibodies [17] are becoming increasingly apparent. Also, the role of epigenetic immunomodulating drugs (such as histone deacetylase inhibitors) in combination with the immune-checkpoint inhibitor anti-Programmed death-1 (anti-PD-1, Nivolumab®) is up for debate. However, mechanisms of these immune responses and responsible antigen structures have to be further elucidated. Since leukemia-associated antigens (LAA) are target structures relevant for the elimination of malignant cells by cytotoxic T lymphocytes (CTL), they represent immunogenic antigens that are candidates for specific immunotherapy.

In fact, in immunotherapy, it is feasible to use combinations of different therapeutic options mostly because their targets differ [18]. However, the impact of an immunomodulating drug and immune checkpoints to attenuate LAA-specific immune responses has not been studied until now. Especially in AML it could be of paramount importance to use specific target antigens [19]. We have previously shown that anti-PD-1 enhanced the cytotoxic effects of LAA-stimulated CTL against leukemic progenitor and colony forming cells (LPC/CFCs). This was most pronounced against nucleophosmin 1 mutated (NPM1^mut^) AML cells when the immunogenic epitope was derived from the mutated region of NPM1 [20]. In this study, we investigated whether different immunotherapeutics alone or in combination with anti-PD-1 can further enhance the LPC/CFC degradation of NPM1^mut^ AML in comparison to AML cells with a wild type NPM1 gene (NPM1^WT^).

Immune checkpoint therapy may have the capacity to reverse immune evasion in myeloid malignancies. While monotherapy with immune checkpoints has produced only modest results, the combination with hypomethylating agents, intensive chemotherapy, and several immune checkpoints have shown promising results [20]. Additionally, these inhibitors act as positive regulators of the immune system and are often used as an initial treatment to improve response rates and increase relapse-free survival after chemotherapy and bone marrow transplantation [21]. Recent research also suggests the potential benefit of immunotherapy in the treatment of AML, specifically targeting CD33, CD123, and CLL-1, as well as the use of immune checkpoints such as anti-PD-1 and anti-cytotoxic T-lymphocyte-associated protein 4 (CTLA4) in the presence or absence of conventional chemotherapy [22]. Single anti-PD-1 monoclonal antibody infusions have shown limited effectiveness in AML [23]. However, in conjunction with hypomethylating agents (HMAs), they represent encouraging treatment options for relapsed/refractory AML patients and for older patients as a first-line treatment [24]. Phase I/II trials with anti-PD-1/PD-L1 antibodies in combination with HMAs have shown encouraging and durable response rates, however there were small patient numbers, the toxicity was quite high, and there were no randomized studies [25].

The aim of this research is to examine ex vivo the potential of using specific T cells with various immunotherapies, either alone or in conjunction with anti-PD-1, to achieve a greater reduction of LPC/CFC, to compare NPM1^mut^ AML to NPM1^WT^ AML cells and to find a feasible way to minimize relapse rates by the use of combination immunotherapies. These results could provide new insights into the emerging field of combination therapies for the treatment of NPM1^mut^ or NPM^WT^ AML.

## Participants and methods

### Sample preparation, isolation and freezing

Peripheral blood samples were received from ten AML NPM1^mut^ and ten AML NPM1^WT^ participants following informed consent and in accordance with the Declaration of Helsinki. The local ethics committee (No. 334/09 and No. 221/14) approved the study protocol. Healthy donor (HD) samples were obtained from the German Red Cross in Ulm. Peripheral blood mononuclear cells (PBMCs) from AML patients and HDs were separated via Ficoll (Pan Biotech, Aidenbach, Germany) density gradient centrifugation, cryopreserved, and stored in liquid nitrogen prior to use. All patient samples consisted of more than 90% leukemic blasts (Table 1).

**Table 1:** Patient information.

Patient No.	Age at TP of sample	Diagnosis	NPM1 ^mut/WT^	Number of leukocytes (x 10^9^/L)
1	73 years	t-AML, complex karyotype	WT	17.6
2	50 years	AML, normal karyotype	WT	8.2
3	61 years	MDS	WT	14.8
4	66 years	AML, no genetic changes	WT	19.8
5	58 years	MDS, 5q-	WT	6.2
6	74 years	Secondary AML, complex karyotype (mono 7), no genetic changes	WT	31.0
7	66 years	AML, normal karyotype	WT	155.2
8	52 years	AML, complex karyotype, refractory	WT	51.5
9	47 years	AML, normal karyotype	mut	9.0
10	76 years	AML, normal karyotype	mut	8.1
11	57 years	AML, FLT3-ITD positive	mut	11.7
12	34 years	AML, normal karyotype	mut	5.2
13	56 years	AML, normal karyotype	mut	8.8
14	70 years	AML, normal karyotype	mut	4.9
15	74 years	AML, normal karyotype, FLT3-ITD positive	mut	134.7
16	64 years	AML, FLT3-ITD negative	mut	4.3
17	51 years	AML, FLT3-ITD positive	mut	4.1
18	54 years	AML, FLT3-ITD negative, FLT3-TKD mutated	mut	5.3
19	75 years	AML, normal karyotype	WT	178.5
20	73 years	AML, complex karyotype	WT	81.2

*FLT3-ITD* FMS-like tyrosine kinase 3-internal tandem duplication, *FLT3-TKD* FMS-like tyrosine kinase 3-tyrosine kinase domain, *MDS* myelodysplastic neoplasm, *t-AML* Therapy related acute myeloid leukemia, *WT* wild type, *mut* mutated, *TP* time point.

### Selection of Leukemia Associated Antigens (LAAs)

To measure the response of specific CTLs, mononuclear cells (MNCs) from AML NPM^WT^ patients were stimulated first with the LAA that generated the largest response against Preferentially expressed antigen in melanoma (PRAME; ALYVDSLFFL) or Wilms Tumor 1 (WT1; RMFPNAPYL), as described in previous analyses [26]. MNCs from NPM1^mut^ patients were stimulated with the mutation related peptide NPM1 (AIQDLCVAV). Only HLA-A2 positive patient samples and HDs were used, since all LAAs were HLA-A2-restricted. The cytomegalovirus (CMV [NLVPMVATV]) peptide served as positive control, data not shown. All peptides were purchased from Proimmune, Oxford, GB.

### Mixed Lymphocyte Peptide Cultures (MLPCs)

PBMC samples from HD were thawed, counted and divided into two equal portions. One fraction served as a source of antigen-presenting cells (APCs), which were irradiated with 30 Gy and pulsed with the respective peptides for 1.5 h at 37 °C, while the other portion was not irradiated and used for the generation of effector T cells with and without the addition of immunotherapeutics. In this way, peptide-specific CD8^+^ allogeneic T cells were generated from HD samples, providing a source of effector (E) cells for further testing. In brief, the appropriate immunotherapeutics were added alone or in combination with the E fraction. Then APCs were mixed with the E cells in a 1:1 ratio. On the second day, IL-2 (2.5 ng/ml) and IL-7 (20 ng/ml) were added and the culture was incubated for eight or nine days and then used for functional testing.

### Addition of immunotherapeutics to cell culture

In line with the results of a former titration [27], 5 µg of the anti-PD-1 antibody (nivolumab) and/or 5 µg of anti-CTLA4 (Ipilimumab; BMS, Munich, Germany)/all-trans retinoic acid (ATRA; Vesanoid, Roche, Basel, Switzerland)/the hypomethylating agent azacitidine (AZA; Vidaza, Celgene, New Jersey, USA) or the immunomodulator lenalidomide (Len; Revlimid, Celgene, Uxbridge, UK) were added on day 0 to the MLPC containing the E fraction alone for one hour, then the irradiated peptide loaded APCs were added to stimulate the MNC fraction. In this way, the direct effects of each immune checkpoint, alone and in combination, on E cells were measured. CD8 + T cells were stimulated with CMV or the respective LAA without an immunotherapeutic as controls.

### Enzyme-Linked-Immuno-Spot (ELISpot)

Membrane flat-bottomed 96 well plates (Merck Millipore Ltd, Carrigtwohill, Ireland) were coated with a solid antibody phase (mAb 1-D1K, 1.5 µg per well). Subsequently, the blocked membranes were incubated with allogeneic pre-stimulated MNCs from MLPC and peptide pulsed blasts from leukemia patients used as APC at a ratio of 5:1. Cytokines bound to the solid antibody phase were visualized with specific antibodies coupled to biotin (mAb 7-B6-1-biotin, 0.1 µg per well), alkaline phosphatase and the corresponding substrate. The evaluation was carried out using an Immunospot ELISpot reader. IFNγ ELISpot (Mabtech, Nacka Strand, Sweden) was performed according to the manufacturer’s instructions.

### Colony-Forming Immunoassays (CFIs)

Allogeneic T cells from healthy donors were taken from MLPCs and used as effector (E) cells at an E:Target (T) ratio of 10:1. Target cells were MNCs that were pulsed with the respective peptide, or pulsed with peptide diluent alone to provide a control. Patient MNCs (without peptide) served as a growth control. After a brief and gentle centrifugation, T and/or E were resuspended in Iscove’s Modified Dulbecco’s Medium (IMDM) containing 2% fetal calf serum (FCS) and added to a 3 mL of hematopoietic stem cells-colony-forming unit (HSC-CFU) complete media with erythropoietin (StemMACS, Miltenyi Biotech, Bergisch Gladbach, Germany) and incubated at 37 °C for 4 h. The media was then aspirated using a syringe. A total of 1.1 mL medium was placed into each cell culture dish (Thermo Scientific, Waltham, MA, USA). Colonies were analysed after a 20-day incubation time; the difference between control and sample in percent was calculated and displayed. A minimum reduction of 10% was noted as being responsive.

### Statistical analysis

Statistical tests were performed using GraphPad PRISM v8. The program was also used to evaluate assays, for comprehensive analysis, for organizing data and for graphing. As a statistical analysis, we used the RM (repeated measure) one-way or Restricted Maximum Likelihood (REML) (mixed-effects model) Analysis of Variance (ANOVA test), * = *p* < 0.05, ** = *p* < 0.01, *** = *p* < 0.001 and **** = *p* < 0.0001.

## Results

**Fig. 1:**
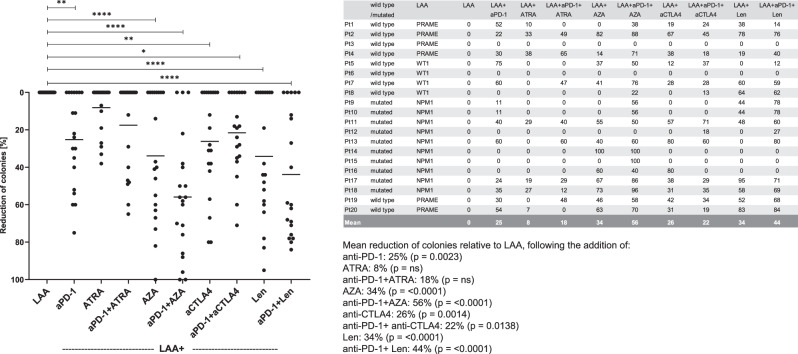
Immune responses by LAA-specific T cells increased after the addition of anti-PD-1. 13 of the 20 patient samples analysed showed a significant reduction in colony numbers when treated with anti-PD-1, with an average reduction of 25% (range 0–75%, *p* = 0.0023). With ATRA and anti-PD-1 and ATRA, the reduction was 8% vs. 18% (respectively, range 0–38% vs. 0–65%, both p = ns). AZA caused a significant reduction in 12 of 20 patient samples of 34% (range 0–100%, *p* < 0.0001) and when using anti-PD-1 and AZA, the decrease in colonies was even higher at 56% (range 0–100%, *p* < 0.0001) in 17 of 20 patient samples. The addition of anti-CTLA4 decreased CFU numbers by 26% (p 0.0014); while anti-PD-1 and anti-CTLA4 decreased them by 22% (*p* = 0.0138). With Len the reduction measured was 34% (range 0–95%, *p *< 0.0001) in 12 of 20 patient samples and with anti-PD-1 and Len in 15 of 20 patient samples with a mean reduction of 44% (range 0–84%, *p* < 0.0001). If only the responders were considered, the reduction rates are even higher for anti-PD-1: 39%, for AZA 57%, for anti-PD-1 and AZA 66%, for Len alone versus anti-PD-1 and Len 57% versus 59%. RM one-way ANOVA * = *p* < 0.05, ** = *p* < 0.01, *** = *p* < 0.001, **** = *p* < 0.0001. aPD-1 anti-programmed death-1, ATRA all-trans retinoic acid, AZA Azacitidine, CTLA4 cytotoxic T-lymphocyte-associated protein 4, LAA leukemia-associated antigen, Len Lenalidomide, NPM1 nucleophosmin 1, PRAME Preferentially expressed antigen in melanoma, Pt patient, WT1 Wilms Tumor 1.

To investigate the effect of the checkpoint-inhibitor anti-PD-1 (nivolumab) alone or in combination with one of four other immunotherapeutics (ipilimumab, Len, ATRA, AZA) we examined the response of allogeneic LAA-specific T cells form healthy donors against leukemic cells and LPC/CFC taken from 20 AML patients (Table 1, Fig. 1). PBMC of HD were stimulated with the LAA that they had showed the strongest response to in former CFI assays to test the above immunotherapeutics alone or in combination with anti-PD-1 [20, 27].

The reduction in colonies (%) was found to be most notable after the addition of anti-PD-1 (anti-PD-1; nivolumab) as a single immunotherapeutic agent. However, using anti-PD-1 and AZA, achieved the largest decrease of any of the dual therapies, in 17 of 20 patient samples.

**Fig. 2:**
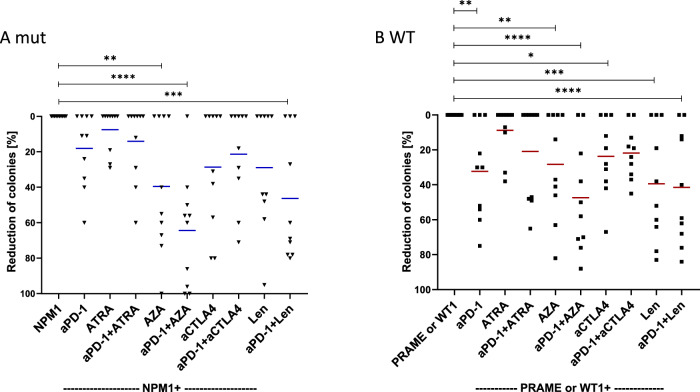
Functional CFI assays were performed with NPM1^mut^ and NPM1^WT^ patient samples. **(A)** Of the ten NPM1^mut^ patients who were tested with anti-PD-1, AZA or anti-PD-1 and AZA in combination, 6, 6 and 9 patients responded; the average reduction rate was 18/40/64 for all and 30/66/72% for responders only. ATRA or anti-PD-1 and ATRA showed a lower reduction rate than anti-PD-1 alone; while the reduction rate for anti-CTLA4 or CTLA4/ anti-PD-1 was 29/21%. Len alone and Len/anti-PD-1 had a significant response of 29/46%. Cells stimulated with LAA only served as a negative control. **(B)** When 10 NPM1^WT^ patient samples were treated with anti-PD-1/AZA/anti-PD-1 and AZA in combination respectively 7/6/8 responded; the average reduction rate was 32/28/47 for all and 46/47/59% for responders only. ATRA/CTLA4 or the respective combinations showed a lower reduction rate than anti-PD-1 alone; Len / anti-PD-1 and Len had a significant response of 39/42%. Stimulated cells with LAA only served as negative control. RM one-way ANOVA * = p < 0.05, ** = p < 0.01, *** = p < 0.001, **** = p < 0.0001. The reduction in the number colonies is shown as a percentage (%) for each figure. aPD-1: anti-programmed death-1; ATRA: all-trans retinoic acid; AZA: Azacitidine; CTLA4: cytotoxic T-lymphocyte-associated protein 4; LAA; leukemia-associated antigen; Len: Lenalidomide; NPM1: nucleophosmin 1; PRAME: Preferentially expressed antigen in melanoma; Pt: patient; WT1: Wilms Tumor 1.

In the ten NPM1^mut^ patient samples, the response rates were most notable for anti-PD-1 and AZA (NPM1 vs NPM1 anti-PD-1 and AZA 64%; p < 0.0001) and even lower in anti-PD-1 and Len (NPM1 vs NPM1 anti-PD-1 and Len 46%; p = 0.0004) (Fig. 2A). Anti-CTLA4 (29%), Len alone (29%) or the combination of anti-PD-1/ anti-CTLA4 (21%) or anti-PD-1/Len (46%). Stimulated cells with LAA only served as negative controls, and were set to 0% reduction. NPM1^WT^ patient samples (Fig. 2B) showed similar relative responses to therapies, with an average reduction of 32% for AZA and 41% for anti-PD-1/AZA in combination while Len and anti-PD-1/Len had a significant response of 39% and 42%, respectively. Stimulated cells with LAA only served as growth control. RM one-way ANOVA * p < 0.05, ** p < 0.01, *** p < 0.001, **** p < 0.0001.

**Fig. 3:**
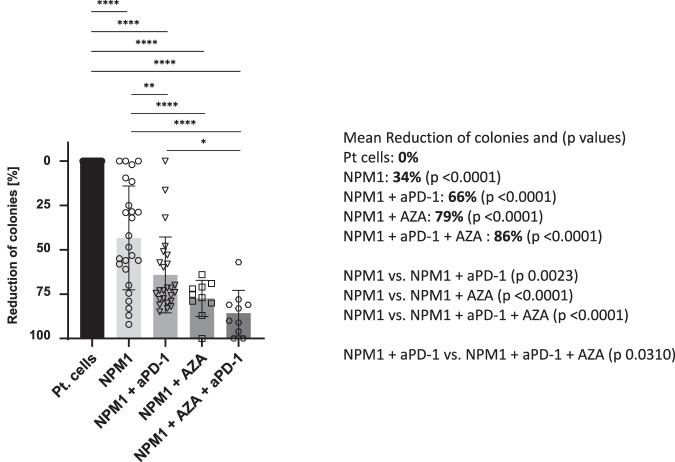
Immune effects of NPM1, anti-PD-1, AZA and the combination of anti-PD-1 and AZA on AML NPM1^mut^ colony numbers. Reductions were all measured compared to colonies formed by the same untreated patient cells. There was a significant reduction in colonies following NPM1^mut^ stimulation in 25 patient samples (34%; p < 0.0001), for NPM1 and anti-PD-1 in 25 patients compared to patient cells alone (66%; p < 0.0001), for NPM1 and AZA in 10 patients (79%; p < 0.0001), and for NPM1 and AZA and anti-PD-1 in 10 patients (86%; p < 0.0001). The comparison of NPM1, with NPM1 and AZA, showed strong significance (p < 0.0001), as did NPM1 with the triple combination of NPM1, AZA and PD-1 (p < 0.0001). REML ANOVA * = p < 0.05, ** = p < 0.01, *** = p < 0.001, **** = p < 0.0001. aPD-1: anti-programmed death-1; AZA: Azacitidine; NPM1: nucleophosmin 1; Pt: patient.

We incubated MNCs from NPM1^mut^ patients with the NPM1 peptide, which were then co-incubated with NPM1 peptide specific T cells from healthy donors and showed that this alone was sufficient to cause a significant reduction in colony numbers by 34% (Fig. 3). This was an extended analysis of samples that we had analysed previously [20, 27] and patient (Pt) cells alone served as a negative control. The addition of allogeneic T cells treated with NPM1 peptide and anti-PD-1 and/or AZA to CFI augmented the reduction of colonies significantly when patient samples were treated with the LAA specific for NPM1^mut^. A significant reduction in the number of colonies was observed with NPM1 and anti-PD-1 (66%), NPM1 and AZA (79%) and NPM1 and anti-PD-1/AZA (86%). There was also significant reduction in the number of colonies when we compared NPM1 with NPM1 and anti-PD-1, with NPM1 and AZA, as well as with the triple combination NPM1 and aPD-1 and AZA. Comparison of NPM1 and PD-1 was only significant with the triple combination NPM1, aPD-1 and AZA.

**Fig. 4:**
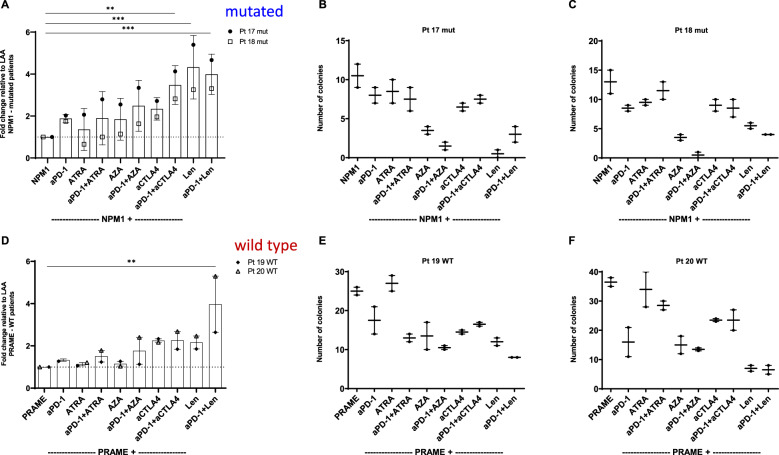
IFNγ production by CD8 + T cells from NPM1^mut^ or NPM1^WT^ patients in the presence of stimulatory LAA. ELISpot results were compared to CFI results for four patients and data were normalized to the respective LAA, which were either mutated NPM1, or for the NPM1^WT^ patients, PRAME. **(A)** The augmented stimulation of the two NPM1^mut^ patients (patients 17 and18) with anti-PD-1 was 2.0/1.7-fold; **(B)** When T cells from AML patient 17 and **(C)** patient 18 were incubated with NPM1^mut^ and immunotherapeutic agents in CFI assays, a correlation between IFNγ production and the reduction in colonies could be seen for anti-PD-1/AZA, Len and anti-PD-1/Len. The stimulation of MNCs from two NPM1^WT^ patients, following normalization to PRAME led to increases in IFNγ levels in the range of 1.3/1.4-fold. Single patient CFU were also used in the ELISpot. In patient 19 and 20 the increased stimulation of T cells **(D)** resulted in a decrease in number of colonies **(E** and **F)**. RM one-way ANOVA * = p < 0.05, ** = p < 0.01, *** = p < 0.001, **** = p < 0.0001. aPD-1: anti-programmed death-1; ATRA: all-trans retinoic acid; AZA: Azacitidine; CTLA4: cytotoxic T-lymphocyte-associated protein 4; LAA; leukemia-associated antigen; Len: Lenalidomide; NPM1: nucleophosmin 1; PRAME: Preferentially expressed antigen in melanoma; Pt: patient; WT1: Wilms Tumor 1.

The production of IFNγ by CD8^+^ T cells from two NPM1^mut^ patients (patients 17 and 18) and two NPM1^WT^ patients (patients 19 and 20) were investigated. All data were normalized to the respective LAA, which was NPM1 for NPM1^mut^ patients and for NPM^WT^ patients, the PRAME peptide. ELISpot results were compared to single CFI results (Fig. 4). Augmented stimulation of the two NPM1^mut^ patients (patient 17 and 18) with anti-PD-1 alone or in combination was 2.0/1.7-fold, respectively (Fig. 4A). The augmented stimulation of the two NPM1^mut^ patients (Patient 17/18) with anti-PD-1 was 2.0/1.7-fold, ATRA 2.1/0.7-fold, anti-PD-1 and ATRA 2.8/1.0-fold, AZA 2.6/1.1-fold, anti-PD-1 and AZA 3.3/1.6-fold, anti-CTLA4 2.7/2.0-fold (p = 0.0036), anti-PD-1 and anti-CTLA4 4.1/2.8-fold, Len 5.4/3.3-fold, respectively (p = 0.0004), or anti-PD-1 and Len 4.7/3.3-fold (p = 0.0009). The results from ELISpot could not always be compared to colony reduction rates, however this was possible with some results. For example, when T cells from patient 17 were incubated with AZA, anti-PD-1/AZA, Len or anti-PD-1/Len in ELISpots there was an increased stimulation of T cells (Fig. 4A) and a reduction in colony numbers (Fig. 4B). Patient 18 with anti-PD-1/AZA, Len and anti-PD-1/Len, showed similar results (Fig. 4A and C). Thus, the effect of stimulating NPM1^mut^ CTL in the presence of immunotherapeutics against LPC/CFC could be demonstrated. When CTL from NPM1^WT^ patients 19 and 20 were stimulated with anti-PD-1 and compared to LAA (PRAME) alone there was a 1.3/1.4-fold increase in IFNγ secretion (Fig. 4D). The other immunotherapeutics alone or in combination with anti-PD-1 showed the following fold changes compared to LAA (PRAME) alone: ATRA 1.1/1.2-fold, anti-PD-1 and ATRA 1.2/1.8-fold, AZA 1.3/1.0-fold, anti-PD-1 and AZA 1.1/2.4-fold, anti-CTLA4 2.3/2.2-fold, anti-PD-1 and anti-CTLA4 1.8/2.7-fold, Len 1.9/2.5-fold, and anti-PD-1 and Len 2.6/5.3-fold (p = 0.0058). The stimulation was lower in the NPM1^WT^ than in the NPM1^mut^ patients. In both patient 19 and 20 (NPM1^WT^ patients) a synergistic effect between anti-PD-1 in combination with AZA could not be seen clearly, and was only observed when a combination of anti-PD-1/Len was used.

**Fig. 5:**
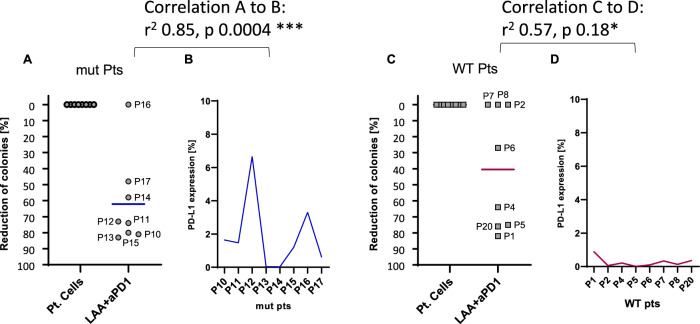
Correlation between the decrease of colonies of patient cells to respective LAA with PD-1 in the CFIs to the PD-L1 expression on patient cells. For the NPM1^mut^ patients **(A and B)** the correlation was stronger with a coefficient of r square 0.85 to 0.57 for NPM1^WT^ patients **(C and D)** (NPM1^mut^ p = 0.0004 vs. NPM1^WT^ p = 0.18). For the NPM1^mut^ patients the peptide NPM1 was used and for the NPM1^WT^ patients, the peptides PRAME or WT-1 were used. The mean decrease in colonies was NPM1^mut^/NPM1^WT^ 62%/41% (pt cells vs. LAA with PD-1) mean PD-L1 expression in flow cytometry NPM1^mut^/NPM1^WT^ 1.9%/0.3%. Two-tailed paired t test * = p < 0.05, ** = p < 0.01, *** = p < 0.001, **** = p < 0.0001. aPD-1: anti-programmed death-1; mut: mutated; Pt/P: patient; WT: wild type.

A correlation was observed between the reduction in colonies formed by patient samples exposed to the respective leukemia-associated antigens (LAAs) with PD-1 in the colony-forming immunoassays (CFIs), and the PD-L1 expression levels measured by flow cytometry in the corresponding patient cells (Fig. 5). This correlation was more notable in NPM1^mut^ patient samples, with a coefficient of determination (r²) of 0.85, compared to an r² of 0.57 in NPM1^WT^ patient samples.

## Discussion

AML is a multifaceted disease with numerous subtypes and complex genetic abnormalities [28]. While treatments have improved in recent years, with remission rates now around 80%, half of patients experience relapse due to the emergence of drug-resistant clones [29].

Bone marrow transplants have been used to treat leukemia patients for around 70 years. Meanwhile, immunotherapy has advanced and now also includes peripheral blood stem cell transplants, donor leukocyte infusions, monoclonal antibodies, adoptive T and/or NK cell therapy, checkpoint blockade and leukemia vaccines [30]. Increasingly, conventional and immunotherapeutic approaches are being used in combination, especially in patients with relapsed and/or refractory disease [31].

In melanoma, as well as other tumor entities, combinations of checkpoint inhibitor therapies showed durable clinical benefits. Wolchok et al reported on the 6.5-year CheckMate 067 results and showed improved outcomes with nivolumab plus ipilimumab or nivolumab versus ipilimumab in patients with advanced melanoma and the benefit of combination over nivolumab monotherapy [32]. In AML the results differ as checkpoint inhibitor monotherapies have shown some clinical effect and improved overall survival in high-risk AML patients not eligible for allogeneic hematopoietic stem cell transplantation [33], while early-phase clinical trials suggest that immune checkpoints and HMAs are safe and more promising [34]. Another difficulty is that HMAs stimulate the immune response against tumor antigens while immune checkpoints are upregulated. In our CFI assays, especially when using NPM1^mut^ AML samples, the results were promising especially when a combination of the checkpoint inhibitor PD-1 and AZA were used. Indeed, we were able to demonstrate a significant reduction in LPC/CFCs in primary AML patient samples in the presence of immune checkpoints and an HMA.

Previously, monotherapy with ATRA was used for the treatment of acute promyelocytic leukemia (APL) with high response rates, but the duration of response was short. Later, the development of ATRA, chemotherapy and arsenic trioxide combinations made APL a highly curable malignancy [35]. We were interested therefore, whether a therapy with ATRA might be feasible for other types of AML, especially for patients with NPM1^mut^. NPM1 mutations may render AML patients susceptible to ATRA, raising the possibility that a strategy targeting mutant oncoproteins to selectively induce NPM1 mutated protein degradation, that involved ATRA, may be a feasible alternative to specific pharmacologic NPM1 inhibitors. This could also be an approach that brings about the selective degradation of the NPM1 mutant proteins [36, 37]. However, our CFI results give an indication that there were no higher responses to ATRA in patients with AML^mut^ compared to AML NPM1^WT^ patients.

The HMAs, AZA and Decitabine, are the standard therapy for patients with higher-risk myelodysplastic neoplasms and for patients with AML, who are not eligible for intensive therapy. Most clinical progress with HMAs has been achieved through the development of pharmacokinetically improved second-generation agents and the search for synergistic drug combinations based on HMAs, has so far been predominantly empirical [38]. There are ongoing attempts to prevent resistance to HMAs through the use of checkpoint inhibitors to boost the immune response [39]. In our CFI, especially for NPM1^mut^ AML samples, the results were very promising suggesting the combination of the checkpoint inhibitor PD-1 with AZA could lead to a very significant reduction in LPC/CFC colonies (86%). The comparison of NPM1 with NPM1 and AZA showed notable significance, as did NPM1 with the triple combination of NPM1, AZA and PD-1.

The difficulty, however, is that while HMAs stimulate the immune response against tumor antigens, the inhibitory immune checkpoints were also upregulated. In our CFI, especially for NPM1^mut^ AML samples, the results were promising as to the combination of the checkpoint inhibitor PD-1 in combination with AZA. We were able to demonstrate a significant reduction of LPC/CFC in primary AML NPM1^mut^ patient samples. We were able to demonstrate a significant reduction of LPC/CFC colonies in primary AML NPM1^mut^ patient samples that likely reflects the re-presentation of the non-native NPM1^mut^ protein to the immune system, by virtue of the use of anti-PD-1 antibodies in combination with AZA. This led to enhanced anti-leukemia responses against NPM^mut^ target proteins compared with NPM^WT^ proteins, which are unlikely to have primed the immune system during early AML development.

CTLA4 as single-agent has shown modest clinical activity in both relapsed/refractory (R/R) AML and MDS. The low mutational burden of AML may be a possible explanation for the lack of activity of T-cell activating immune checkpoints, especially CTLA-4 and PD-1 inhibitors [14, 15]. We saw a moderate but solid reduction in stem cell-like cells with the checkpoint inhibitors CTLA4 or PD-1 alone, and again we saw that the combination of these immunotherapeutics did not enhance these anti-leukemia effects [14, 27].

The immunomodulatory drug Len has anti-inflammatory, anti-proliferative, pro-apoptotic and anti-angiogenic properties and in this way promotes anti-tumor immunity. The effects of Len differ from those of other compounds used to treat AML, making Len an interesting agent for use in AML and in combination with existing agents [18].

The issue of addressing resistance mechanisms in targeted therapy is another crucial consideration. The adaptability of cancer cells is significant because of clonal evolution [40]. In the development of AML, somatic mutations accumulate in hematopoietic stem/progenitor cells, resulting in uncontrolled growth [41]. This ultimately leads to the development of resistance mechanisms, evasion of the immune system and continued proliferation of leukemia cells [42].

Taken together, we have shown that combinations of immunotherapeutic approaches increase antigen-specific immune responses against leukemic cells but also LPC/CFC, especially the combination of LAA peptides with the anti-PD-1 antibody and one further immunomodulating drug, like AZA, could be an interesting option for further clinical trials and might open up interesting application possibilities, potentially as an further building block in the rapidly developing field of combination therapies in AML.

## Data Availability

The data generated during and/or analysed during the current study are available from the corresponding author on reasonable request.
